# Gender‐specific effects of raising Year‐1 standards on medical students' academic performance and stress levels

**DOI:** 10.1111/medu.14068

**Published:** 2020-03-20

**Authors:** Karen M. Stegers‐Jager, Mesut Savas, Jeroen van der Waal, Elisabeth F. C. van Rossum, Andrea M. Woltman

**Affiliations:** ^1^ Institute of Medical Education Research Rotterdam Erasmus MC University Medical Centre Rotterdam Rotterdam the Netherlands; ^2^ Division of Endocrinology Department of Internal Medicine Erasmus MC University Medical Centre Rotterdam Rotterdam the Netherlands; ^3^ Department of Public Administration and Sociology Erasmus University Rotterdam Rotterdam the Netherlands

## Abstract

**Context:**

Medical schools are challenged to create academic environments that stimulate students to improve their study progress without compromising their well‐being.

**Objectives:**

This prospective comparative cohort study investigated the effects of raising Year‐1 standards on academic performance and on students' chronic psychological and biological stress levels.

**Methods:**

In a Dutch medical school, students within the last Bachelor's degree cohort (n = 410) exposed to the 40/60 (67%) credit Year‐1 standard (67%‐credit cohort) were compared with students within the first cohort (n = 413) exposed to a 60/60 (100%) credit standard (100%‐credit cohort). Main outcome measures were Year‐1 pass rate (academic performance), mean score on the Perceived Stress Scale (PSS, psychological stress) and hair cortisol concentration (HCC, biological stress).

**Results:**

Year‐1 pass rates were significantly higher in the 100%‐credit cohort (odds ratio [OR] 4.65). Interestingly, there was a significant interaction effect (OR 0.46), indicating that raising the standard was more effective for male than for female students. PSS scores (n = 234 [response rate [RR]: 57%] and n = 244 [RR: 59%] in the 67%‐ and 100%‐credit cohorts, respectively) were also significantly higher in the 100%‐credit cohort (*F*_(1,474)_ = 15.08, *P* < .001). This applied specifically to female students in the 100%‐credit cohort. Levels of HCC (n = 181 [RR: 44%] and n = 162 [RR: 39%] respectively) did not differ between cohorts, but were significantly higher in female students (*F*_(1,332)_ = 7.93, *P* < .01). In separate models including cohort and gender, both PSS score (OR 0.91) and HCC (OR 0.38) were significantly associated with Year‐1 performance. Only students with both high PSS scores and high HCC values were significantly at risk of lower Year‐1 pass rates (OR 0.27), particularly male students.

**Conclusions:**

Raising the Year‐1 performance standard increased academic performance, most notably in male students. However, it also increased levels of perceived stress, especially in female students. In particular, the combination of high levels of perceived stress and biological stress, as measured by long‐term cortisol, was related to poor academic performance. The study suggests a relationship between raising performance standards and student well‐being, with differential effects in male and female students.

## Abstract 2

Raise the bar and students will meet it, but at what cost? Stegers‐Jager et al. show that raising performance standards yield improved passing rates, but also drives stress.

**Key message**

Raising Year‐1 performance standards leads to higher Year‐1 pass rates, but also increases levels of perceived stress in medical students, with differential effects in male and female students.

## 1 INTRODUCTION

The challenge for medical schools worldwide is to create academic environments that stimulate students to improve their study progress,^1^ without compromising their health.^2^ The urge to seek measures to improve student progress is driven by the substantial investment in students made by both the students themselves and society.^3^, ^4^ A possible strategy for achieving this involves the implementation of academic dismissal policies that require students to make satisfactory study progress.^1^ Failure to meet set standards leads to significant delay in a student's progress (eg, in systems in which students are unable to proceed to the subsequent year if they fail to achieve the required credits, such as in year classes) or academic dismissal. Academic dismissal policies are common at universities in the USA and have been applied at Dutch universities for the last two decades. However, the literature on academic dismissal policies is scarce and the limited evidence regarding their impact on study progress is inconclusive.^5^, ^6^ Furthermore, policy interventions shown to be effective in some schools have proved unsuccessful in other disciplines,^1^, ^5^ and their effectiveness depends on characteristics of the student population.^5^ Additionally, although data on the possible side‐effects of these policy interventions are lacking, there is increasing fear that such measures imply a cost to student well‐being.^7^

The introduction of an academic dismissal policy that required students to obtain at least two‐thirds of the total number of Year‐1 credits was found not to affect dropout, completion and study rates during the first 2 years of medical school, but was accompanied by higher rates of attendance at support sessions.^1^ The lack of effect on study progress may be explained by the fact that an academic dismissal policy focuses on minimum standards rather than on the benefits of an optimal study rate. This raises the question of what might happen if the minimum requirements were to be set to the maximum, or, in other words, if students were expected to obtain all Year‐1 credits within 1 year.^8^ To the best of our knowledge, the impacts of a stricter dismissal policy on student well‐being and academic performance in general, and within medical school more specifically, remain unknown.

Studies have found high prevalences of distress amongst medical students in comparison with age‐matched controls including non‐medical student peers,^9^, ^10^ which hampers learning, interferes with professional development and, in the long term, affects personal well‐being and patient care.^11^ Previous research has shown that not only student‐related factors, such as gender, but also school‐related factors, such as evaluation or grading systems and learning environments, affect student distress^12^ and consequently influence student well‐being.^13^ An important issue concerns whether there is an optimum level of stress for academic performance. Whereas acute stress may have some metabolic, immunological and cognitive benefits, chronic stress may cause cognitive decline, adverse effects in the hippocampus, and increase the risk for neurodegenerative disease, as well as cardiometabolic disease.^14^, ^15^ To date the scarce research in medical students has focused mainly on acute perceived stress and less on biological stress.^12^ Additionally, the methods used previously to measure levels of cortisol, the main stress hormone (eg, in blood, urine and saliva) are complicated by the circadian rhythm and pulsatile process of cortisol secretion, and by the influence of acute stress. Therefore, little is known about the relationship between chronic stress and academic performance. Current models of emotions, based on appraisal processes, emphasise the individualistic way in which people respond to stressful circumstances.^13^ An individual's responses (psychological and biological) to demands (eg, the difficulty of an examination) that threaten an important goal (eg, becoming a doctor or a lawyer) are highly dependent on that individual's perceptions of the demands and the resources (eg, student characteristics) that person has available to meet those demands. Given the high prevalences of distress amongst medical students in comparison with their age‐matched controls,^9^, ^10^, ^16^, ^17^ it is vital to gain understanding of how the (increased) use of academic dismissal policies relates to stress and performance amongst students. In view of the differential individual responses to stressful circumstances, we consider it crucial to also take student characteristics into account. More specifically, we will look at differences between the genders as a recent review suggested that female medical students tend to experience higher levels of stress invoked by assessment than male students, although this finding was not consistent across all studies.^12^ Our basic claim is that for a proper understanding of the impacts of implementing academic dismissal policies, the potential for these policies both to positively affect academic outcomes and to induce chronic stress, and consequently a decline in student well‐being and academic outcomes, must be investigated. It is, therefore, imperative to take both academic outcomes *and* student stress levels into account in order to uncover the impact and relevance of academic progress policies.

The present study investigated the effects of raising Year‐1 standards on academic performance and on students’ chronic psychological and biological stress levels. The changes in policy at our medical school offered us the rare opportunity to respond to calls for research that compares differential effects for assessments with different stakes (high and even higher^18^), has relatively long follow‐up durations and looks at the long‐term effects of ongoing exposure to assessment.^12^ In this study, we used a relatively novel parameter by measuring cortisol concentrations in scalp hair because these reflect the long‐term cortisol levels of recent months. This method has been well validated.^19^ We and others have shown that this method provides a unique opportunity to reliably measure the biological effects of stressful circumstances in humans (cf. Groeneveld et al,^20^ Staufenbiel et al,^21^ Stalder et al^22^).

We aimed to answer the following research questions: (a) What are the effects of raising Year‐1 standards on academic performance and on medical students' chronic perceived and biological stress levels?, and (b) Is there a differential effect for male and female students?

## 2 METHODS

### 2.1 Context

The present study was carried out at the Erasmus MC Medical School in Rotterdam, the Netherlands. The first year of the integrated and theme‐oriented Bachelor curriculum at this school is composed of thematic blocks and competence‐based learning lines for which students can obtain a maximum of 60 credits under the European Credits Transfer System. From 2005 the Erasmus MC Medical School implemented an academic dismissal policy whereby substandard progress resulted in academic probation (at 12 months) or academic dismissal (at 24 months) (Table 1). Until 2014, students whose progress was substandard (ie, students who achieved less than 40 credits) at 12 months were allowed to repeat Year 1 (probation), whereas students with 41‐59 credits at 12 months were allowed to engage in Year‐2 modules alongside their remaining Year‐1 module(s). Credits were awarded for each module provided the student obtained a sufficient grade (ie, ≥ 5.5 out of a maximum of 10.0) on the examination. In 2014, the Year‐1 credit standard was raised from 67% (40/60 credits) to 100% (60/60 credits). Students were required to achieve an average grade of at least 6.0 on the nine examinations, but two grades of 5.0‐5.49 were allowed under the condition that they were not obtained in the same thematic block. The intention of raising the standard was to increase the academic progress of Bachelor students.^8^ Students who failed to earn the required number of credits at the end of the first year (12 months) were not allowed to repeat Year 1 but were immediately subject to academic dismissal. The change in the assessment policy was the only major curriculum alteration in recent years.

**Table 1:** Academic probation and dismissal policies

Time from enrolment, months	Type of action		Standard (maximum)
	67%‐credit cohort^a^	100%‐credit cohort^b^	
12	Academic probation		<40 credits (60)
12		Academic dismissal^c^	<60 credits (60)
24	Academic dismissal^c^		<60 credits (120)

a Lowest grade allowed: 5.5, minimum grade point average (GPA).

b Two grades of 5.0‐5.49 were allowed, minimum GPA: 6.0.

c Dispensation possible for 1 year for temporary personal circumstances.

### 2.2 Participants and procedure

Participants in this study were students in two consecutive cohorts, which included the last cohort to be subject to the requirement to obtain 67% of credits (entering in 2013, 67%‐credit cohort) and the first cohort to be subject to the requirement to obtain 100% of credits (entering in 2014, 100%‐credit cohort) and comprised 410 and 413 students, respectively. In order to collect data on psychological stress, all students in both cohorts were invited to complete a survey at 1.5 months before the final Year‐1 examination, which is taken in early July. Students were recruited during a single large‐scale lecture and online. To determine average biological stress levels during the last 3 months of the academic year, scalp hair samples were collected from student volunteers in both cohorts on the last day of the academic year. Students were recruited immediately after completing their final examination. We deliberately planned to administer the survey on psychological stress in the middle of the 3‐month period covered by the hair samples.

Data on academic performance were derived from the university student administration system and confidentiality was guaranteed. As data were collected as part of regular academic activities and only aggregate data are reported, individual consent was not necessary. For the measures of psychological and biological stress, written informed consent was obtained from all participants and confidentiality was guaranteed. Students were able to participate voluntarily and were not given incentives for participation. Prior to the analyses, all data were coded and saved without direct identification information. The current study was carried out in accordance with the Declaration of Helsinki and was deemed exempt from review after evaluation by the Medical Ethics Committee of Erasmus MC University Medical Centre Rotterdam.

### 2.3 Outcome measures

#### 2.3.1 Perceived stress

The Perceived Stress Scale (PSS) questionnaire^23^ consists of 14 items assessing both general distress and inability to deal with stress. Example items are: ‘In the last month, how often have you felt nervous and stressed?’ and ‘In the last month, how often have you felt that you could not cope with all the things that you had to do?’ Items are scored on a 5‐point Likert scale (0 = never; 4 = very often). Higher scores reflect a higher level of perceived stress (total score range: 0‐56). We used a validated Dutch version of this questionnaire.^21^

#### 2.3.2 Biological stress

To assess biological stress levels, we collected scalp hair samples from the posterior vertex. From each hair sample, the 3 cms most proximal to the scalp was analysed to provide data on average cortisol exposure in the preceding 3 months. Cortisol was extracted from scalp hair using methanol and hair cortisol concentration (HCC) was measured using an enzyme‐linked immunosorbent assay (ELISA) kit (DRG Instruments GmbH, Marburg, Germany) as described previously.^24^ Additionally, students completed a questionnaire on hair‐related factors that could potentially affect cortisol concentration, such as hair colour, washing frequency, use of corticosteroids during the previous 6 months, other medication use and distressing life events (herein referred to as the ‘hair questionnaire’). Hair cortisol values were log‐transformed to normalise the distribution.

#### 2.3.3 Academic performance

The academic performance indicator used in this study was the Year‐1 curriculum pass rate, which was defined as the proportion of students in each cohort who earned all 60 credits in the Year‐1 curriculum within 12 months after enrolment.

### 2.4 Statistical analysis

To enable valid comparisons, the 67%‐ and 100%‐credit cohorts were compared on the pre‐admission variables of gender, using chi‐squared tests, and on age and pre‐university education grade point average (pu‐GPA), using analyses of variance (ANOVAs). Pre‐university GPA represented the mean grade obtained by a student during the final year of pre‐university education. Final pu‐GPAs were based half on school examinations and half on the national examination. Additionally, the cohorts were compared on the different variables measured in the hair questionnaire using chi‐squared tests.

We first conducted exploratory analyses comparing the 67%‐ and 100%‐credit cohorts and male and female students on the three outcome measures. Differences in percentages were tested using chi‐squared tests and differences in means using Student's *t*‐test. As measures of effect size, we included odds ratios (ORs) (values of 1.22, 1.86 and 3.00 represent small, medium and large effects, respectively) or inverse equivalents (values of 0.82, 0.54 and 0.33 represent small, medium and large effects, respectively)^25^ and Cohen's d (values of 0.20, 0.50 and 0.80 represent small, medium and large effect sizes, respectively).^26^

Next, we used logistic regression to calculate an OR for the effect of the academic dismissal policy (67%‐credit versus 100%‐credit requirement) on Year‐1 pass rate. Statistical interaction terms were used to study the potentially differential effects of the academic dismissal policy by gender. We included ORs as measures of effect size.^25^ We used a two‐way ANOVA to examine the effect of the academic dismissal policy and gender on PSS sum scores and on HCC values. Generalised omega‐squared was computed as a measure of effect size as recommended by Olejnik and Algina,^27^ with values of 0.01, 0.06 and 0.14 indicating small, medium and large effects, respectively.

Finally, we used logistic regression to test three models: (a) a model including the academic dismissal policy, gender and PSS; (b) a model including the academic dismissal policy, gender and HCC, and (c) a model including the academic dismissal policy, gender and a compound score based on median values for PSS score and HCC. The compound score divided participants into four groups: (a) LowLow (≤ median for both PSS score and HCC value); (b) HighLow (> median for PSS score and ≤ median for HCC value; (c) LowHigh (≤ median for PSS score and > median for HCC value), and (d) HighHigh (> median for both PSS score and HCC value). All variables were entered simultaneously in a multivariable logistic regression model.

Analyses were performed in spss Version 21.0 (IBM Corp., Armonk, NY, USA). A *P*‐value of <.05 was considered to indicate differences of statistical significance.

## 3 RESULTS

### 3.1 Student characteristics

The PSS was completed by the majority of the students (67%‐credit cohort, n = 234 [57%]; 100%‐credit cohort, n = 244 [59%]). All respondents answered all items on the questionnaire. With respect to biological stress, we collected scalp hair samples from 181 students in the 67%‐credit cohort (44%) and from 162 students in the 100%‐credit cohort (39%). All of these students also completed the hair questionnaire.

The 67%‐ and 100%‐credit cohorts did not show significant differences with respect to gender (66% female and 67% female, respectively), mean age (19.27 years and 19.26 years, respectively) and pu‐GPA (7.15 and 7.16, respectively). The only significant difference on the hair questionnaire was a higher score in the 100%‐credit cohort for distressing life events, most of which referred to examinations as indicated by the students (41% and 71% in the 67%‐ and 100%‐credit cohorts, respectively; χ^2^_(1)_ = 30.25, *P* < .001; OR 3.47, 95% confidence interval [CI] 2.21‐5.45).

### 3.2 Academic performance

The exploratory analyses showed significantly higher Year‐1 pass rates in the 100%‐credit cohort compared with the 67%‐credit cohort, both for the total cohorts and for men and women separately (Table 2). Female students had a significantly higher Year‐1 pass rate than male students (Table 3).

**Table 2:** Academic performance and stress measures in the 67%‐ and 100%‐credit cohorts

	Cohort				Statistics		
	67%‐credit		100%‐credit				
	n	%	n	%	χ^2^	*P*‐value	ES^a^
Year‐1 completion^b^							
Total	203	49.5	302	73.1	48.38	<.001	2.77
Male	53	37.9	102	73.9	36.62	<.001	4.65
Female	150	55.6	200	72.7	17.48	<.001	2.13

	n	Mean	n	Mean	*t*	*P*‐value	ES^c^
PSS							
Total	234	24.10	244	27.82	−4.93	<.001	0.45
Male	68	22.91	75	24.47	−1.03	.31	—
Female	166	24.59	169	29.31	−5.65	<.001	0.62
HCC							
Total	181	23.78	162	22.65	0.60	.55	—
Male	67	21.03	66	19.41	0.49	.62	—
Female	114	25.57	96	25.19	0.18	.86	—

Abbreviations: ES, effect size; HCC, hair cortisol concentration; PSS, Perceived Stress Scale.

a Odds ratio.

b Percentage of all students from initial cohort.

c Cohen's d.

**Table 3:** Academic performance and stress measures in male and female students

	Gender				Statistics		
	Male		Female				
	n	%	n	%	χ^2^	*P*‐value	ES^a^
Year‐1 completion^b^	155	56	350	64	5.56	<.05	1.42

	n	Mean	n	Mean	*t*	*P*‐value	ES^c^
PSS	143	23.72	335	26.97	3.72	<.001	0.38
HCC	133	20.21	210	25.39	2.51	<.05	0.26

	n	%	n	%	χ^2^	*P*‐value	ES^a^
Compound score							
PSS low HCC low	35	39	45	25	8.68	<.05	1.88
PSS high HCC low	14	15	43	24			—
PSS low HCC high	25	28	41	23			—
PSS high HCC high	16	18	49	28			—

Abbreviations: ES, effect size; HCC, hair cortisol concentration; PSS, Perceived Stress Scale.

a Odds ratio.

b Percentage of all students from initial cohort.

c Cohen's d.

**Figure 1:**
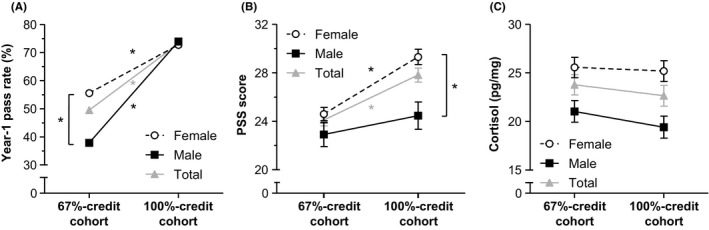
Year‐1 performance and stress outcomes in study cohorts of medical students tasked with achieving 67% and 100% of Year‐1 credits, respectively. A, Year‐1 pass rates in the total 67%‐credit (n = 410) and 100%‐credit (n = 413) cohorts, and separately in each cohort for female (n = 270 and n = 275, respectively) and male (n = 140 and n = 138, respectively) students. B, Mean ± standard error (SE) scores on the Perceived Stress Scale (PSS) for all participants in the 67%‐credit (n = 234) and 100%‐credit (n = 244) cohorts, and for female (n = 166 and n = 169, respectively) and male (n = 68 and n = 75, respectively) students. C, Mean ± SE untransformed hair cortisol concentration (HCC) in all participants in the 67%‐credit (n = 181) and 100%‐credit (n = 162) cohorts, and in female (n = 114 and n = 96, respectively) and male (n = 67 and n = 66, respectively) students. Statistical analyses were performed to show differences between cohorts (total or subgroup) or between male and female students within a cohort. *P < .05

The logistic regression analysis revealed that Year‐1 pass rates were significantly higher in the 100%‐credit cohort (Wald χ^2^_(1)_ = 34.77, *P* < .001; OR 4.65, 95% CI 2.79‐7.75) and in female students (Wald χ^2^_(1)_ = 11.39, *P* < .001; OR 2.05, 95% CI 1.35‐3.11). Furthermore, a significant interaction effect (Wald χ^2^_(1)_ = 6.00, *P* < .05; OR 0.46, 95% CI 0.25‐0.86) indicates that raising the standard was more effective for male than for female students (Figure 1A).

### 3.3 Stress

Students in the 100%‐credit cohort scored significantly higher on the PSS than students in the 67%‐credit cohort (Table 2). Sub‐analyses by gender revealed that only female students had significantly higher PSS scores in the 100%‐credit cohort compared with the 67%‐credit cohort. In general, female students had significantly higher PSS scores than male students (Table 3).

In line with these findings, the two‐way ANOVA revealed significantly higher PSS scores in the 100%‐credit cohort (*F*_(1,474)_ = 15.08, *P* < .001, ω_G_^2^ = 0.03) and in female students (*F*_(1,474)_ = 16.29, *P* < .001, ω_G_^2^ = 0.03). There was no significant interaction effect (*F*_(1,474)_ = 3.84, *P* = .051, ω_G_^2^ = 0.01) (Figure 1B).

Students in the two cohorts did not significantly differ in HCC values (Table 2). However, female students had higher HCC levels than male students (Table 3). These findings were confirmed by the two‐way ANOVA. Hair cortisol concentrations did not differ between the cohorts (*F*_(1,343)_ = 0.33, *P* = .57), but were significantly higher in female students (*F*_(1,343)_ = 7.55, *P* < .01, ω_G_^2^ = 0.02); there was no interaction effect (*F*_(1,343)_ = 0.15, *P* = .69) (Figure 1C).

### 3.4 Stress and academic performance

**Figure 2:**
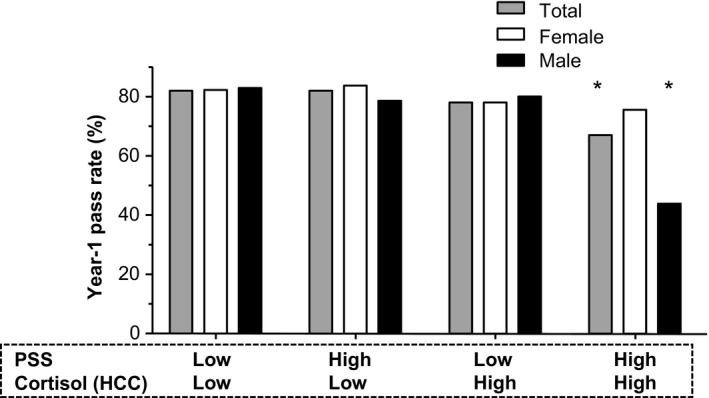
Year‐1 performance and compound Perceived Stress Scale (PSS) scores and hair cortisol concentration (HCC) values. Year‐1 pass rates for all participants combined (n = 268) and by gender (ie, female [n = 178] and male [n = 90]) divided according to compound score based on median values for PSS (26.00) and HCC (25.30). Reference group: LowLow (≤ median for both PSS score and HCC value). *P < .05

Both PSS and HCC data were available for 135 students (33%) in the 67%‐credit cohort and for 133 students (32%) in the 100%‐credit cohort. There was no significant correlation between PSS score and HCC (*r*_(268)_ = .11, *P* = .07). In separate models including cohort and gender, both PSS (Wald χ^2^_(1)_ = 33.35, *P* < .001; OR 0.91, 95% CI 0.89‐0.94) and HCC (Wald χ^2^_(1)_ = 4.17, *P* < .05; OR 0.39, 95% CI 0.16‐0.96) were significantly associated with Year‐1 performance. Only students with high values (above median) on both the PSS and HCC were significantly at risk of lower Year‐1 academic performance (Wald χ^2^_(1)_ = 9.22, *P* < .01; OR 0.26, 95% CI 0.11‐0.62), particularly male students (Figure 2). Notably, the gender proportion was not comparable within the four compound score groups (χ^2^_(3)_ = 8.68, *P* < .05) as male students were more likely to have both low PSS scores and low HCC values than female students (39% and 25%, respectively; OR 1.88) (Table 3).

## 4 DISCUSSION

This study shows that raising the Year‐1 performance standard increased academic performance, most prominently in male students. However, it also increased levels of perceived stress, especially in female students. There was no effect on levels of biological stress as measured by long‐term cortisol secretion. Nevertheless, the combination of high perceived stress and high biological stress was found to be related to poor academic performance.

It is not surprising that Year‐1 performance improved after the Year‐1 standard was raised because this is in line with findings in previous studies that have shown superior performance on tests with higher stakes^28^, ^29^, ^30^ (ie, higher consequences of performance) or with higher performance standards^31^, ^32^ (ie, higher demands in order to pass). However, it is not in line with previous findings by ourselves and others on the effectiveness of implementing academic dismissal policies.^1^, ^5^, ^6^, ^33^ An important difference between the current and these previous studies is that the present study is the first to investigate the effect of setting the minimum standard to be equivalent to the maximum. To date, two possible explanations have been suggested for the limited effects of academic dismissal policies on medical student performance: (a) a threshold effect, which assumes that students may reduce their efforts after obtaining the minimum number of credits required, and (b) a ceiling effect based on the assumption that there is little room for improvement given the already high Year‐1 pass rates of medical students.^8^ Our study suggests that the first explanation is more plausible because some students were apparently able to improve their study progress after they were (strongly) encouraged to do so.

A striking finding concerned the gender‐related difference in the effectiveness of the measure and the observation that male students were able to surpass female students in Year‐1 performance. It is possible that the threshold effect applies more to male than to female students. Previously, it has been suggested that despite the importance of intrinsic motivation, external triggers (ie, higher performance standards) may have a powerful additional effect on academic motivation.^18^ Our data suggest that this additional effect may be stronger for male than for female students. This is in line with findings in previous studies, which have shown that male students tend to have higher extrinsic or controlled motivation and lower intrinsic or autonomous motivation than female students.^34^, ^35^

The increased academic performance coincided with increased levels of perceived stress, especially in female students. Higher levels of assessment stress or anxiety in female students than in male students have been reported previously, but this gender effect was not consistent across the studies included in the review by Lyndon et al.^12^ One possible explanation for the gender‐related differences in perceived stress refers to personality traits, of which the combination of neuroticism and conscientiousness in particular has been found to be more commonly present in female medical students and to be associated with higher levels of stress.^36^ Other potential explanations for the gender‐related differences in perceived stress refer to previously identified gender‐based differences in levels of overestimation^37^ and of rumination.^38^ Despite the increase in perceived stress brought about by the implementation of the new policy, our students generally reported lower levels of perceived stress than medical students in the USA^39^ and Pakistan.^40^

Raising the standard did not have an effect on levels of biological stress. However, students who scored highly on both stress outcomes, particularly male students, showed worse study performance. This finding emphasises the individual approaches of students in evaluating their well‐being during medical school. Furthermore, differences in dynamics between psychological and biological stress may explain the non‐significant relationship between the two stress outcomes. Future studies may want to investigate the differential consequences of high levels of both biological and psychological stress in male and female students.

The current study has several strengths and limitations that should be mentioned. A first strength is that we included a rather large sample size in both cohorts, which increased the power to identify differences and allowed us to perform multiple group comparisons. Nevertheless, it may be that our subsamples were not representative of the total cohorts. However, we do not have any reason to suspect differences in non‐participants between the two cohorts. Focusing on the participants from whom we collected hair samples revealed no significant differences with respect to gender, mean age and pu‐GPA between the two cohorts, and similar conclusions with respect to academic performance and perceived stress. Another strength of our study is that the students were well characterised for both individual parameters at admission and different stress parameters at the end of Year 1. An important limitation is that no data were collected on stress‐related psychological and physical effects, which makes it difficult to infer anything about the consequences of higher levels of stress in this population. Additionally, it is not possible to infer causality on the basis of our data, despite the fact that data on stress and academic performance were collected at different time‐points. To ascertain definitive causal pathways, further studies that measure stress levels throughout the first year are required. Although the groups were quite similar regarding pre‐admission variables and the 100%‐credit cohort more frequently reported examination‐related life events, the use of historical control subjects in the study design prevents us from drawing definitive conclusions about the effects of the academic progress policy on the outcomes.

This study has some practical implications for medical schools that aim to improve their students' progress and offers some directions for future research. First, our findings suggest a relationship between the raising of performance standards and student well‐being. As we noted earlier, an important aspect of the relationship between stress and academic performance relates to the issue of whether there is an optimum level at which students can perform best. In this study, we found that an increased academic demand was associated with better performance, as well as relatively higher PSS scores, reflecting psychological stress levels in the past month, whereas no differences were found in average long‐term biological stress experienced over the preceding 3 months. Despite the use of measurements to detect chronic stress, it remains uncertain whether the higher levels of perceived stress observed were present during the whole of the first year and continued into the second year. This is of particular importance given the relatively high frequencies of depression, as well as suicidal thoughts, in medical students.^41^ Therefore, we recommend that medical schools monitor their students’ stress levels when implementing measures to increase study progress and consider implementing interventions to improve student well‐being, such as wellness programmes that teach mind‐ and body‐based stress reduction skills and formal faculty advisor/mentor programmes for small groups.^2^

Second, our study revealed gender‐related differences in the effects of the raising of standards. This suggests that changes in the academic environment may have differential effects in male and female students. Therefore, as in medical practice, we urge medical educationalists to take differential effects in subgroups into account, both in designing and implementing, and in evaluating the effects of educational innovations. This may be particularly important for educational innovations that influence feelings of autonomy. Generally, autonomous motivation is reported to be associated with greater psychological well‐being than controlled motivation.^42^ Further research is required to explore possible gender‐based differences in that pattern, especially in an academic environment.

## 5 CONCLUSIONS

Raising the Year‐1 performance standard increased academic performance, most prominently in male students. However, it also increased levels of perceived stress, especially in female students. In particular, the combination of a high level of perceived stress and a high level of biological stress was related to poor academic performance. Our study suggests a relationship between the raising of performance standards and student well‐being, with differential effects in male and female students. Medical schools should take these differences into account when trying to strike a balance between optimising study progress and supporting student well‐being.

## AUTHOR CONTRIBUTIONS

KMS‐J, EFCvR and AMW substantially contributed to the conception and design of the study. KMS‐J analysed the data and wrote the first draft of the article. All authors (KMS‐J, MS, JvdW, EFCvR and AMW) collected and interpreted the data, revised it critically for important intellectual content, approved the final manuscript for publication, and have agreed to be accountable for all aspects of the work in ensuring that questions related to its accuracy or integrity are appropriately investigated and resolved.

## CONFLICTS OF INTEREST

No competing interests.

## ETHICAL APPROVAL

The current study was carried out in accordance with the Declaration of Helsinki and was deemed exempt from full review after evaluation by the Medical Ethics Committee of Erasmus MC University Medical Centre, Rotterdam.
